# Correction: Kapranova et al. Essential Trace Elements in the Shells of Commercial Mollusk Species from the Black Sea and Their Biotechnological Potential. *Animals* 2025, *15*, 1637

**DOI:** 10.3390/ani15182642

**Published:** 2025-09-09

**Authors:** Larisa L. Kapranova, Juliya D. Dikareva, Sergey V. Kapranov, Daria S. Balycheva, Vitaliy I. Ryabushko

**Affiliations:** A.O. Kovalevsky Institute of Biology of the Southern Seas of RAS, 2 Nakhimov Ave., Sevastopol 299011; lar_sa1980@mail.ru (L.L.K.); dikareva.julija@rambler.ru (J.D.D.); sergey.v.kapranov@yandex.ru (S.V.K.); rabushko2006@yandex.ru (V.I.R.)

**Error in Affiliation**

In the original publication [1], there was an error regarding the affiliation address. The authors would like to correct the affiliation to: “A.O. Kovalevsky Institute of Biology of the Southern Seas of RAS, 2 Nakhimov Ave., Sevastopol 299011”.

The authors state that the scientific conclusions are unaffected. This correction was approved by the Academic Editor. The original publication has also been updated.
